# A Similar Secretome Disturbance as a Hallmark of Non-pathogenic *Botrytis cinerea* ATMT-Mutants?

**DOI:** 10.3389/fmicb.2019.02829

**Published:** 2019-12-06

**Authors:** Amélie de Vallée, Pascal Bally, Christophe Bruel, Lucie Chandat, Mathias Choquer, Cindy Dieryckx, Jean William Dupuy, Sophie Kaiser, Marie-Pascale Latorse, Elise Loisel, Géraldine Mey, Guillaume Morgant, Christine Rascle, Julia Schumacher, Adeline Simon, Eytham Souibgui, Muriel Viaud, François Villalba, Nathalie Poussereau

**Affiliations:** ^1^Microbiologie, Adaptation et Pathogénie, UMR 5240, Univ Lyon, Université Lyon 1, Bayer SAS, Lyon, France; ^2^UMR BIOGER, INRA, AgroParisTech, Université Paris-Saclay, Thiverval-Grignon, France; ^3^Centre de Recherche La Dargoire, Bayer SAS, Lyon, France; ^4^Plateforme Protéome, Centre de Génomique Fonctionnelle, Université de Bordeaux, Bordeaux, France; ^5^Federal Institute for Materials Research and Testing (BAM), Berlin, Germany

**Keywords:** *Botrytis cinerea*, *Agrobacterium tumefaciens*-mediated transformation, pathogenicity, secretomics, transcriptomics

## Abstract

The gray mold fungus *Botrytis cinerea* is a necrotrophic pathogen able to infect hundreds of host plants, including high-value crops such as grapevine, strawberry and tomato. In order to decipher its infectious strategy, a library of 2,144 mutants was generated by random insertional mutagenesis using *Agrobacterium tumefaciens-*mediated transformation (ATMT). Twelve mutants exhibiting total loss of virulence toward different host plants were chosen for detailed analyses. Their molecular characterization revealed a single T-DNA insertion in different loci. Using a proteomics approach, the secretome of four of these strains was compared to that of the parental strain and a common profile of reduced lytic enzymes was recorded. Significant variations in this profile, notably deficiencies in the secretion of proteases and hemicellulases, were observed and validated by biochemical tests. They were also a hallmark of the remaining eight non-pathogenic strains, suggesting the importance of these secreted proteins in the infection process. In the twelve non-pathogenic mutants, the differentiation of infection cushions was also impaired, suggesting a link between the penetration structures and the secretion of proteins involved in the virulence of the pathogen.

## Introduction

The gray mold fungus *Botrytis cinerea* is a widely distributed pathogen that can infect more than 500 genera of plants including high-value crops, such as grapevine, tomato, strawberry or ornamental flowers. All the parts of the plants such as leaves, fruits, flowers or roots can be colonized and destroyed by this pathogen (Elad et al., 2016). Each year, this ascomycete causes important pre and post-harvest damages leading to important economic losses. *B. cinerea* is considered to be a typical necrotrophic fungus and has become a model to study fungal infection (Dean et al., 2012). This fungus secretes an arsenal of toxins, including organic acids and secondary metabolites, and a set of numerous hydrolytic enzymes that contribute to degrade host tissues and sustain the fungal nutritional needs during colonization. To precise the molecular mechanisms involved in the infection process, forward genetic approaches were broadly performed. In parallel, random mutagenesis based on *Agrobacterium tumefaciens*-mediated transformation (ATMT) was developed to select mutants altered in virulence and to identify the responsible genes. In the case of the highly aggressive *B. cinerea* strain B05.10, a first collection of 2,367 mutants was constructed leading to 68 mutants that showed significant reduction in virulence on tomato and bean plants (Giesbert et al., 2012). The identification of the transfer DNA (T-DNA) insertion site revealed that integrations were mostly single copy in non-coding regions as previously described for other fungal ATMT libraries (Betts et al., 2007; Blaise et al., 2007; Münch et al., 2011). The characterization of several mutants and the validation by targeted inactivation of the identified genes led to the identification of new virulence-related proteins such as BcPPA2A (catalytic subunit of a protein phosphatase), BcSPT3 (subunit of the Spt-Ada-Gcn5-Acetyl-transferase (SAGA) complex) (Giesbert et al., 2012), BcSEP1 (formin required for nucleation of F-actin) (Giesbert et al., 2014), BcLTF1 (light-responsive transcription factor 1) and BcKDM1 (putative histone 3 lysine 36/lysine 9 (H3K36/K9) demethylase) (Schumacher et al., 2019). In 2017, another T-DNA library was constructed and used to identify a kinesin (KLP-7)-encoding gene involved in virulence (Tayal et al., 2017). Finally, Liu et al. (2018) used the same approach to show that the key gluconeogenic gene *Bcpck1* encoding the phosphoenolpyruvate carboxykinase could be considered as a virulence-associated gene. More recently, the screening of the same ATMT library identified a *B. cinerea*-specific virulence-associated gene (*Bchbf1*) participating to the differentiation of penetration structures such as infection cushions (Liu et al., 2019). Considering that the number of virulence-related genes already described in *B. cinerea* increases regularly and that the last annotation of the genome of *B. cinerea* describes 11,701 genes with 30% of unknown functions (van Kan et al., 2017), we can assume that the generation and characterization of more mutants will identify additional novel virulence-associated factors providing new insights in the infection process of *B. cinerea*. For these reasons, we followed up on this random mutagenesis approach and generated a new set of 2,144 ATMT strains.

In this work, we report on the study of twelve non-pathogenic mutants on several hosts. They carry single T-DNA insertions in different loci. Phenotypic analyses were performed, and common deficient traits were observed between the different mutants, in particular a defect in the differentiation of infection cushions (ICs), a fungal penetration structure. Proteomics and transcriptomics analyses were performed for 4 mutants and revealed a common profile of reduced lytic enzyme activities in comparison to that of the parental strain B05.10.

## Materials and Methods

### Culture Media

Twelve media were used, containing 1.5% agar for solid media. (1) TNK-hyg70 [derived from Tanaka-B medium (Ou, 1985)]: 1% glucose, 0.2% yeast extract, 0.2% NaNO_3_, 0.2% KH_2_PO_4_, 0.05% MgSO_4_⋅7H_2_O, 0.01% CaCl_2_⋅2H_2_O, 0.0004% FeSO_4_⋅7H_2_O, microelements as in Tanaka-B, 70 μg/mL hygromycin B (InvivoGen), pH 5.5. (2) MA-hyg70c: 2% malt extract, 70 μg/mL hygromycin B, 200 μM cefotaxim (Sigma), pH 5.5. (3) CM (Complete Medium) as described in Pontecorvo et al. (1953). (4) MM (Minimal Medium) as described in Schumacher et al. (2014). (5) Spo (Sporulation medium): 0.5% glucose, 2% malt extract, 0.1% bacto-peptone, 0.1% casein hydrolyzate, 0.1% yeast extract, 0.02% sodium pH 5.0. (6) Specific-substrates: 316 mg/L Gamborg B5 medium (Duchefa), 0.5% agarose, 0.5% carboxy-methyl-cellulose (Sigma) or polygalacturonic acid (Sigma) or xylan (Sigma) or D-galacturonic acid (Sigma) or 2% glucose (Sigma) or D-xylose (Sigma), pH 5.5. (7) BSA: 0.5% bovine serum albumin (Sigma), 0.5% agarose, pH 5.5. (8) AA (Amino Acids): 0.05 mg/mL tyrosine, 2 mg/mL arginine, histidine, methionine, 4 mg/mL lysine, tryptophan, 5 mg/mL threonine, 6 mg/mL isoleucine, leucine, phenylalanine, 15 mg/mL valine, 0.01 mg/mL adenosine, 0.04 mg/mL uracil, 0.5% agarose, pH 5.5. (9) CCPX: 400 mg/L Gamborg B5 medium, 0.1% carboxy-methyl-cellulose, 0.1% casein (Sigma), 0.1% polygalacturonic acid, 0.1% xylan (Roth), pH 5.5. (10) Cucumber medium: 15 one-week-old cucumber cotyledons in 1 mM CaCl_2_, 1 mM MgCl_2_, 1 mM NaH_2_PO_4_, 25 mM KCl, microelements (0.1 μM CoCl_2_⋅6H_2_O, 0.1 μM CuCl_2_⋅5H_2_O, 0.1 mM FeNa-EDTA, 49 μM H_3_BO_3_, 4.5 μM KI, 60 μM MnCl_2_⋅H_2_O, 1 μM Na_2_MoO_4_⋅2H_2_O, 7 μM ZnCl_2_⋅7H_2_O) and vitamins (0.56 mM myo-inositol, 8 μM nicotinic acid, 5 μM pyridoxine-HCl, 30 μM thiamine-HCl). (11) PDB1/4: potato dextrose broth diluted to the fourth. (12) LB: 1% bactopeptone, 0.5% yeast extract.

### Strains and Culture Conditions

Strain B05.10 of *B. cinerea* [teleomorph *Botryotinia fuckeliana* (de Bary) Whetzel] was used as a recipient strain for genetic modifications (Quidde et al., 1998). B05.10 and transformed strains were maintained on Spo medium as previously described (Antal et al., 2012), supplemented when necessary with hygromycin B (70 μg/mL). Mycelial plugs (4-mm diameter) served as inocula and the cultures were incubated in the dark at 21°C. For DNA preparations, mycelium was grown on solid Spo medium with cellophane membranes for 3 days. Strain LBA1126 of *A. tumefaciens* carrying the pBHt2 plasmid (Mullins et al., 2001) was maintained at 28°C on LB medium supplemented with spectinomycin (250 μg/mL) (Sigma).

### Generation of *B. cinerea* Mutants by ATMT

The *B. cinerea* transformant library was generated using *A. tumefaciens*-mediated transformation (Rolland et al., 2003). Incorporation of the T-DNA carrying a hygromycin resistance cassette into the fungal genome allowed selection of the transformants on solid TNK-hyg70 medium and subsequent isolation of individual clones on solid MA-hyg70c medium. Most primary transformants presumably are heterokaryons (Rolland et al., 2003), therefore elimination of the parental nuclei was attempted. To this end, two rounds of single spore isolation and subculturing under selective pressure were performed when the transformants produced conidia.

### Southern Blot, TAIL-PCR and Rescue-PCR Analysis

Southern blot analyses were performed as described previously (Rolland et al., 2003) with the following adjustments. Fungal genomic DNA was isolated using the DNeasy plant mini kit (Qiagen), digested with *Eco*RV or *Nco*I and hybridized with a *hph* DIG-probe using the PCR DIG Probe Synthesis Kit (Roche) and primer pair hph15/hph21 (Supplementary Table S1). Digested DNA was transferred onto a nylon membrane and treated following manufacturer’s instructions using DIG Luminescent Detection Kit (Roche). The DIG signal was captured with ChemiDoc XRS camera (Bio-Rad).

Identification of the insertion sites was performed by TAIL-PCR as described in Liu et al. (1995), using the Silverstar Taq polymerase (Eurogentec) and pBHT2-specific primers (Mullins et al., 2001). The final PCR products were sequenced at the INRA-BIOGER genotyping platform using a CEQ8000 capillary sequencing system (Beckman Coulter).

For rescue-PCR, genomic DNA was digested by *Eco*RV or *Xho*I (NEB). After enzyme inactivation, T4 DNA ligase (NEB) was added for 3 days of ligation reaction at 4°C. The genomic sequences flanking the T-DNA were amplified from the generated circularized DNA by using primers targeting the 5′ and 3′ ends of the T-DNA (Supplementary Table S1). Two successive PCR reactions were performed using nested primers with Herculase II polymerase (Agilent). The final amplicons were purified from agarose gels using the Monarch DNA gel extraction kit (NEB) and sequenced.

### Pathogenicity Assays and Phenotypic Analyses

Infection assays were performed using 1-week-old cucumber cotyledons (*Cucumis sativus*), primary French bean leaves (*Phaseolus vulgaris* var Saxa) and wounded apple fruit (var “Goldrush”). The plants were inoculated with 4-mm agar plugs collected from 3-day-old cultures (Spo medium) and placed in 7.5 μl GB5 medium supplemented with 2% glucose. Infected plants were incubated at 21°C under 80% relative humidity and dark-light (16 h/8 h) conditions. Symptoms were scored up to 7 days post inoculation (dpi) and more than three independent biological replicates were assessed.

Radial growth, conidiation, medium acidification and formation of sclerotia were measured as described previously (Rascle et al., 2018). Fungal growth experiments on specific-sugar, BSA and AA media were performed in 6-well plates with 2-mm agar plugs collected from 3-day-old MM cultures. Growth was measured at 3–4 dpi by monitoring the colonized surfaces using a ZEN Macroscope plus 2012 blue edition software (Zeiss). Conidia were counted under a microscope after 17 days of incubation on Spo medium. For each test, three independent biological experiments and statistical analysis (Student’s *t*-test) were performed. Infection cushions were observed by reverse microscopy 2 days after inoculation of agar plugs (2-mm) in 50 μl PDB 1/4 in 24-well plate (Thermo Scientific).

### Activities of Secreted Enzymes

CCPX medium (50 ml) was inoculated with 10 mycelial plugs (4-mm) from 3-day-old Spo cultures. The cultures were incubated under shaking (110 rpm) at 21°C in the dark for 7 days. Mycelia were discarded by centrifugation (10 min, 1000 *g*, 4°C) and aliquots of culture supernatants were frozen in liquid nitrogen and kept at −80°C before being used as samples in the following enzymatic reactions. Xylanase and cellulase activities were recorded on a plate reader TECAN infinite M1000 using the EnzChek-Ultra-xylanase assay kit (Thermofisher) and the cellulase assay kit (Abcam), respectively, according to the manufacturer’s instructions. The activities were determined from standard reactions with purified enzymes (xylanase from *Trichoderma longibrachiatum* (Sigma, X2629) and cellulase from *Trichoderma reesei* (Serva)). Polygalacturonase activity was determined by incubating (30 min, 48°C) samples (200 μl) and substrate (100 μl polygalacturonic acid 5 mg/mL) in 100 μl acetate buffer 60 mM, pH 4.0. Reactions were stopped by addition of 1 ml TBC (0.1 M Na-tetraborate, 0.1 M boric acid, 0.1% cyanoacetamide) and heating (100°C) for 10 min. Free galacturonic acid was detected by spectrophotometry at 270 nm (Molecular Devices Spectramax-485) and quantitated from a galacturonic acid standard curve. Acid protease activity was measured by incubating (24 h, 30°C) samples (150 μl) with 450 μl of 1% hemoglobin (Sigma), pH 3.5. Reactions were stopped with 25% trichloroacetic acid (400 μl). After centrifugation, supernatants were mixed with 0.5 M NaOH (vol/vol) in UV microplates and optical density was read at 280 nm. Lipase activity was measured after 2 h at 37°C using 50 μl samples and the Sigma lipase assay kit (MAK046). Laccase activity was measured by incubating 10 μl of samples and 30 μl of ABTS as substrate (Sigma) in 230 μl of 50 mM Na-acetate buffer, pH 4.0. Oxidation of ABTS was recorded at 405 nm (Molecular Devices Spectramax-485) during 30 min at 30°C. One unit was defined as the amount of enzyme producing an increase of 0.01 OD units per min. All activities were measured on three independent biological replicates.

### Production of Reactive Oxygen Species and Response to Oxidative Stress

Identical quantities of fresh mycelia from 3-day-old cellophane cultures were incubated for 2 h in 3,3′-diaminobenzidine (DAB) staining solution as described previously (Schumacher et al., 2014). Sensitivity to oxidative stress was determined by measuring radial growth after 72 h on solid complete medium supplemented with 10 mM H_2_O_2_ or 500 μM menadione (Schumacher et al., 2014).

### Proteomic Analysis

The exoproteome was prepared as previously described except that 200 ml of CCPX liquid medium were inoculated with ten 4-mm agar plugs. The steps of sample preparation and protein digestion were performed as described in Rascle et al. (2018). The analysis was performed by the proteomics core facility at University of Bordeaux^1^.

NanoLC-MS/MS analyses were performed using an Ultimate 3000 RSLC Nano-UPHLC system (Thermo Scientific, United States) coupled to a nanospray Orbitrap Fusion^TM^ Lumos^TM^ Tribrid^TM^ Mass Spectrometer (Thermo Fisher Scientific, California, United States). Each peptide extracts were loaded on a 300 μm ID × 5 mm PepMap C18 precolumn (Thermo Scientific, United States) at a flow rate of 10 μL/min. After a 3 min desalting step, peptides were separated on a 50 cm EasySpray column (75 μm ID, 2 μm C18 beads, 100 Å pore size, ES803, Thermo Fischer Scientific) with a 4–40% linear gradient of solvent B (0.1% formic acid in 80% ACN) in 55 min. The separation flow rate was set at 300 nL/min. The mass spectrometer operated in positive ion mode at a 2.0 kV needle voltage. Data was acquired using Xcalibur 4.1 software in a data-dependent mode. MS scans (m/z 375-1500) were recorded at a resolution of *R* = 120000 (@ m/z 200) and an AGC target of 4 × 10^5^ ions collected within 50 ms, followed by a top speed duty cycle of up to 3 s for MS/MS acquisition. Precursor ions (2 to 7 charge states) were isolated in the quadrupole with a mass window of 1.6 Th and fragmented with HCD@30% normalized collision energy. MS/MS data was acquired in the ion trap with rapid scan mode, AGC target of 3 × 10^3^ ions and a maximum injection time of 300 ms. Selected precursors were excluded for 60 s.

Sequest HT and Mascot 2.4 algorithms through Proteome Discoverer 1.4 Software (Thermo Fisher Scientific Inc) were used for protein identification in batch mode by searching against Ensembl *B. cinerea* database (12 060 entries, release 31). Two missed enzyme cleavages were allowed. Mass tolerances in MS and MS/MS were set to 10 ppm and 0.6 Da. Oxidation of methionine, acetylation of lysine and deamidation of asparagine and glutamine were searched as dynamic modifications. Carbamidomethylation on cysteine was searched as static modification. Peptide validation was performed using Percolator algorithm (Käll et al., 2007), and only “high confidence” peptides were retained corresponding to a 1% False Positive Rate at peptide level. The mass spectrometry proteomics data have been deposited to the ProteomeXchange Consortium via the PRIDE (Deutsch et al., 2017) partner repository with the dataset identifier PXD013559.

Raw LC-MS/MS data were imported in Progenesis LC-MS QI 2.0 (Non-linear Dynamics Ltd., United Kingdom) for feature detection, alignment, and quantification. All sample features were aligned according to retention times by manually inserting up to one hundred landmarks followed by automatic alignment to maximally overlay all the two-dimensional (m/z and retention time) feature maps. Singly charged ions and ions with higher charge states than six were excluded from the analysis. All remaining features were used to calculate a normalization factor for each sample to correct for experimental variation. Peptide identifications (with *p* < 0.01, see above) were imported into Progenesis. For quantification, all unique peptides of an identified protein were included, and the total cumulative abundance was calculated by summing the abundances of all peptides allocated to the respective protein. ANOVA as performed at the protein level and filtered to identify features with *p* < 0.05. Quantitative data were considered for proteins quantified based on a minimum of two unique peptides.

### RNAseq Analysis

For RNAseq analysis, 2 × 30 ml liquid Spo medium were inoculated with finely sliced mycelia grown on Spo medium in the dark. Following 44 h incubation (21°C, 100 rpm), the mycelia were collected by centrifugation (3000 rpm, 5 min), washed twice in sterile water and used to inoculate cucumber medium (30 ml). The mycelia were harvested by centrifugation (4000 rpm, 10 min, 4°C) after 48 h of incubation (21°C, 100 rpm), frozen in liquid nitrogen, lyophilized and ground. Total RNA was extracted from 4 mg of ground material using the RNeasy midi kit (Qiagen) according to the manufacturer’s instructions. Traces of genomic DNA were removed by DNase treatment (Ambion) and RNA quality was assessed using the Bioanalyzer RNA 6000 Nano kit (Agilent). cDNA single-end libraries were prepared and sequenced (50 bp) on a HiSeq2500 Illumina platform at ViroScan-ProfileXpert (France). In total, 19 libraries were sequenced (four biological replicates for B05.10, NP/T1, NP/T2, NP/T3, and 3 replicates for NP/T4). The quality of the reads was checked using FastQC^2^. 14 to 22.5 million reads were obtained for each library. Reads were mapped to the *B. cinerea* B05.10 reference genome (van Kan et al., 2017) using STAR 2.5 (Dobin et al., 2013). 98.5 to 99% of reads were uniquely mapped to the genome. Normalization and differential gene expression analyses were performed with the Bioconductor package DESeq2 (Love et al., 2014) using SARTools facilities (Varet et al., 2016). Genes with a base mean of at least 50 normalized reads, a corrected *p-*value < 0.05, and more than a two-fold change in transcript level were considered as significantly DE. Details on the experiments, raw and normalized values are available in the Gene Expression Omnibus Database (GEO)^3^ under the entry GSE134112.

For proteomics and transcriptomics data analyses, functional enrichment Gene Ontology (GO) analyses were performed using gProfiler (Reimand et al., 2007; Raudvere et al., 2019) and FungiFun2 (Priebe et al., 2015) with the significance tests supplied by both tools, i.e., tests based on hypergeometric distribution with a corrected *p*-value threshold at 0.05. Putative SPs without TM were predicted with SIGNALP (Almagro Armenteros et al., 2019). The classification of CAZy was extracted from the CAZy database^4^ (Lombard et al., 2014) and the substrate specificity determined by Van den Brink and de Vries (2011), Glass et al. (2013), and this study.

## Results

### Insertional Mutagenesis in *B. cinerea* and Screening for Non-pathogenic Transformants

**FIGURE 1:**
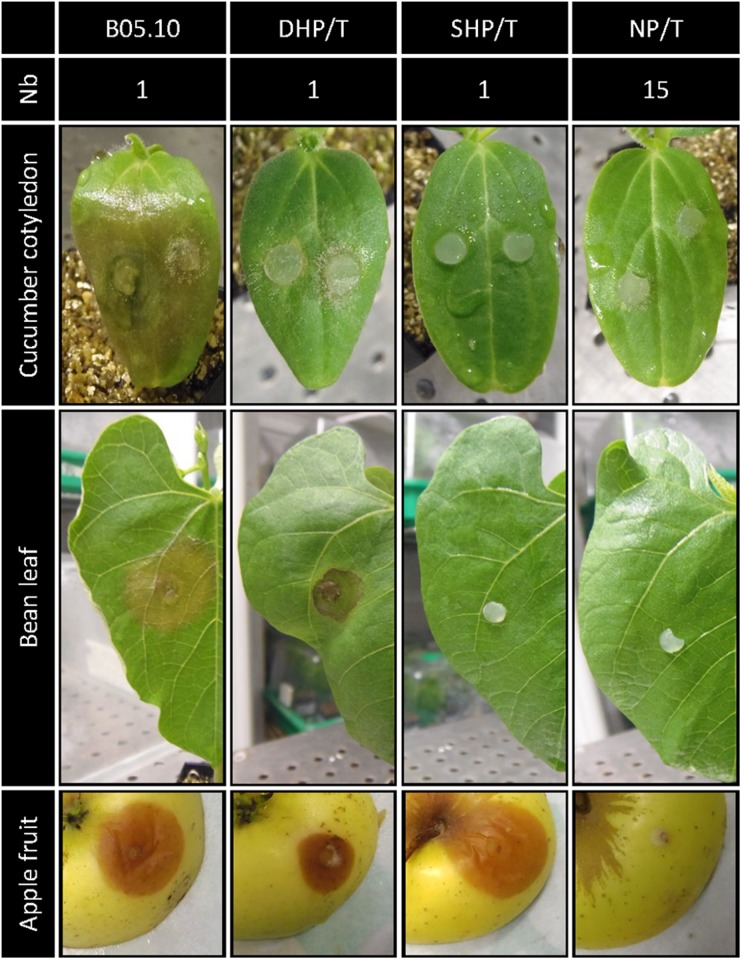
Selection of non-pathogenic transformants of *B. cinerea*. The virulence of the *B. cinerea* ATMT transformants was compared to that of the parental strain B05.10 on cucumber cotyledons (*C. sativus*, top), primary leaves of French bean (*P. vulgaris*, middle) and wounded apple fruits (var goldrush, bottom). Agar explants of non-conidiating mycelia were used as inoculum and pictures were taken 4 days post inoculation (dpi). Three independent experiments (≥48 inoculation points) were performed. Nb, number of transformants exhibiting identical phenotypes; DHP/T, double-host pathogenic transformant; SHP/T, single-host pathogenic transformant; NP/T, non-pathogenic transformant.

*Botrytis cinerea* B05.10 was mutagenized via an *A*. *tumefaciens* strain carrying a hygromycin resistance cassette flanked by the T-DNA left and right border sequences. Following 12 transformation experiments 2,144 hygromycin-resistant transformants were collected. Southern blot analysis of 186 randomly chosen transformants revealed that 77% carried a single insertion of the T-DNA in the genome. This high percentage is similar to those described in the first collection of *B. cinerea* (Giesbert et al., 2012) or in other fungal ATMT libraries (Blaise et al., 2007; Choi et al., 2007; Münch et al., 2011). With the aim of detecting non-pathogenic strains, cucumber cotyledons were inoculated with young mycelia (3-day-old) of the transformants and the parental strain B05.10. Among the 1,691 ATMT mutants analyzed, 65 revealed an alteration of the infection process on cucumber cotyledons, 44 exhibited a significant attenuated virulence and 17 of them were non-pathogenic. (1% of 1,691 screened mutants). To strengthen this result, these 17 transformants were challenged with two other hosts, namely intact bean leaves and wounded apple fruits. Of the 17 transformants tested, one infected both bean leaves and apple tissues (DHP/T), one was only virulent on apple fruit (SHP/T) and 15 were non-pathogenic on all three hosts (NP/T) (Figure 1 and Supplementary Figure S1). These 15 non-pathogenic transformants were subjected to Southern blot analysis and 12 of them showed a hybridization profile corresponding to a single integration of the T-DNA in their genome (Supplementary Figure S1). These 12 non-pathogenic transformants (NP/T1-12) were selected for further phenotypic studies.

### Growth and Developmental Characteristics of the Non-pathogenic Strains

**FIGURE 2:**
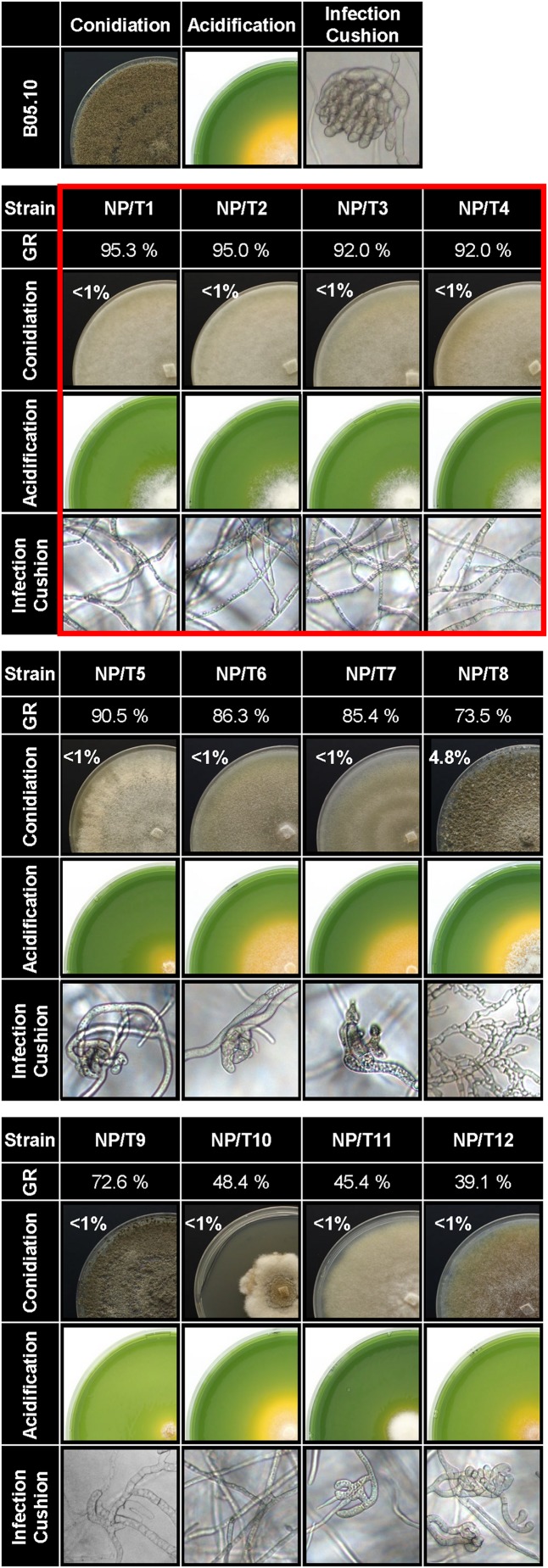
Phenotypic characterization of the 12 non-pathogenic transformants. B05.10 strain and the transformants NP/T1-12 were grown for 4 days on solid complete medium. Their radial growth rates (GR) were calculated as a percentage of that of the parental strain B05.10 [raw data are presented in Supplementary Table S7 (*n* = 3)]. Conidiation was quantified from 17-days-old cultures grown on sporulation medium under blacklight conditions. Results are expressed as a percentage of the conidia produced by the parental strain in the same conditions [6.10^7^ ± 3.10^7^ spore/ml (*n* = 3)]. Solid complete medium adjusted to pH 8.0 and supplemented with bromophenol blue (yellow at pH < 6.0) was used to monitor acidification at 4 dpi. Agar explants of mycelia were used as inocula. Infection cushions (ICs) were observed by inverted microscopy after 48 h of incubation in liquid PDB1/4 medium on plastic surfaces. Photos and data are representative of three independent experiments. Red frame, selected strains for further studies.

The success of the infection relies, at least in part, on the formation of penetration structures for entering the plant tissues (Choquer et al., 2007) and on hyphal growth for efficient colonization and degradation of the plant tissues. Besides, production of organic acids by the fungus plays a role in virulence (Rascle et al., 2018). Whether these fungal capabilities were affected in the 12 non-pathogenic transformants was therefore explored (Figure 2 and Supplementary Table S7). On solid rich medium, the radial growth rate was slightly (<10% of that of the parental strain), moderately (13–30%) and strongly (50–60%) reduced in 5, 4, and 3 transformants, respectively. Under appropriate conditions for conidiation, the pigmentation of the colonies varied, but the production of conidia was drastically reduced in all transformants when compared to the parental strain (<5% in NP/T8 and <1% in the 11 remaining NP/T). On complete medium adjusted to pH 8.0, 5 of the 12 transformants (NP/T1-4 and NP/T11) were deficient in acidifying their environment. On plastic surfaces, infection cushions (ICs) were absent in 7 NP/T or partially developed in the remaining 5, and no evolution could be seen with time (data not shown). Lastly, we noticed that none of the transformants produced sclerotia even after 3 weeks of culture in the dark (data not shown). Altogether, these results highlighted production of conidia, infection cushions and sclerotia as common deficient traits in the 12 non-pathogenic transformants. Of these 12 strains, 4 (NP/T1-4) exhibited regular hyphal growth rates and failed to acidify the culture medium (Figure 2 red box). These four strains were selected for more detailed characterization.

### Comparative Analysis of the Exoproteomes of the NP/T1-4 Strains

As secretion of lytic enzymes is assumed playing an important role in plant infection by *B. cinerea*, the exoproteome of transformants NP/T1-4 was explored through a shotgun proteomic analysis. To promote enzyme secretion, the transformants and the parental strain were grown in a synthetic liquid medium containing polysaccharides and casein as nutrients (CCPX medium). Following mass spectrometry analysis, a total of 1,719 proteins were identified in the growth medium collected at the end of the culture (Supplementary Table S2). Spectral counting was used to sort the proteins whose peptides occurred differentially (*p-*value < 0.05). The analysis was restricted to the proteins identified with a minimum of two unique peptides and a fold change above 2 between the parental strain and the transformants. This led to the listing of 964 proteins that were considered up-accumulated (649 proteins) or down-accumulated (315 proteins) in at least one of the 4 transformants when compared to the parental strain. Noticeably, contradictory data (up-accumulation in one transformant and down-accumulation in another one) was limited to two proteins (Supplementary Tables S2, S3).

Based on the predictions of SP (Almagro Armenteros et al., 2019) and TM, the 964 extracellular proteins were divided into 320 proteins putatively secreted through the conventional pathway (presence of a SP and absence of a TM) (Supplementary Table S3) and 644 proteins whose presence in the medium would derive from secretion through non-conventional pathways [absence of SP (614) or presence of a TM (30)] (Supplementary Table S3).

**FIGURE 3:**
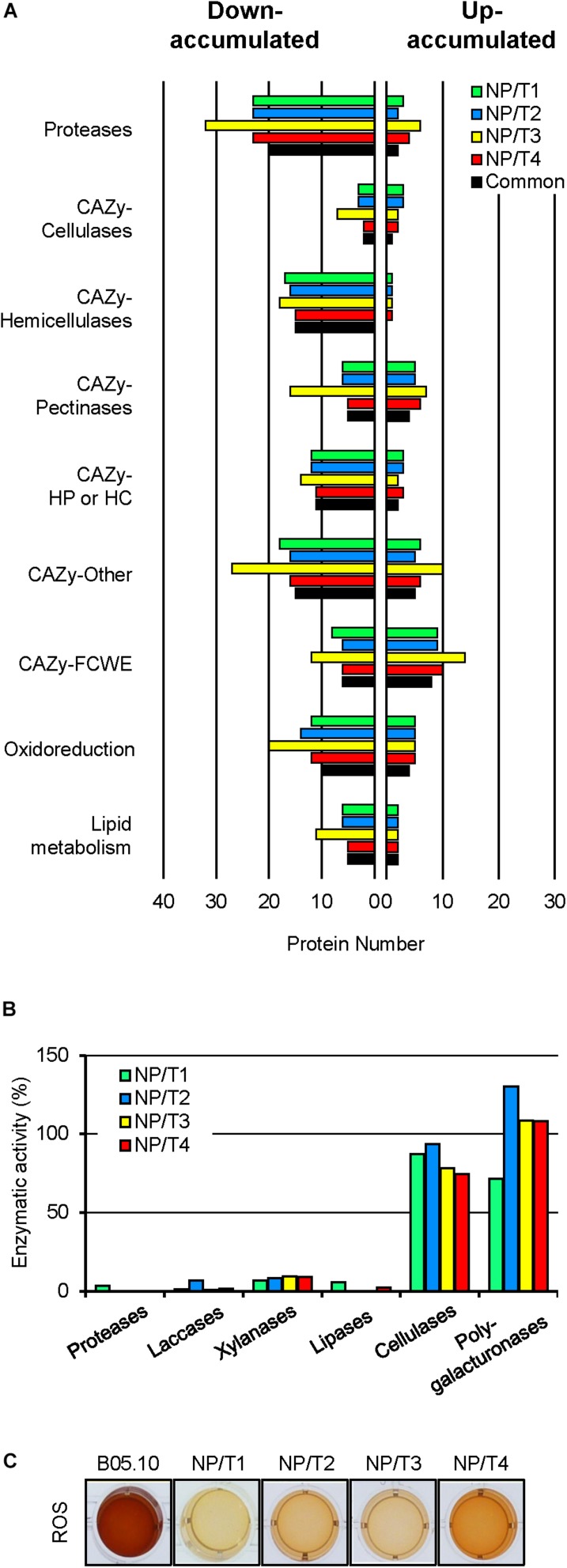
Exoproteome shotgun analysis, enzymatic activities and H_2_O_2_ production of transformants NP/T1-4. **(A)** Functional categories classification of predicted extracellular proteins [SPs but no TM (see Supplementary Table S3)], that were down- or up-accumulated in comparison to the parental control (B05.10) (fold change >2, *p*-value < 0.05) for the 4 transformants NP/T1-4. Common proteins detected in all transformants NP/T1-4 are indicated in black. Exoproteome analyses were performed after 7 days of growth in liquid CCPX medium (three independent biological experiments). CAZy, carbohydrate active enzymes; FCWE, fungal cell wall enzymes; HP, Hemicellulose Pectin; HC, Hemicellulose Cellulose. **(B)** Enzymatic activities measured in CCPX medium at 7 dpi for B05.10 strain and the transformants NP/T1-4. The results of 3 independent reactions are shown as percentages of the parental strain (B05.10) values. Details are available in Supplementary Table S6. **(C)** Production of H_2_O_2_, visualized by the oxidation of soluble colorless DAB into a brown precipitate 2-h post-incubation (hpi). ROS, reactive oxygen species.

Of the 320 proteins putatively trafficking through the conventional secretion pathway, 222 were down-accumulated and 98 were up-accumulated in at least one transformant. When compared to the genome-based theoretical list of predicted proteins with a SP in *B. cinerea*, a gene ontology (GO biological process) analysis revealed no GO-term enrichment for the up-accumulated proteins, but the enrichment of « proteolysis » and « carbohydrate metabolic process » for the down-accumulated proteins (data not shown). Further *in silico* analysis led to a more detailed functional classification for 245 of the 320 proteins (Supplementary Table S3). As shown in Figure 3A, more than 20 proteases were down-accumulated in the transformants. Multiple CAZy targeting plant cell wall components, in particular hemicellulose, were also down-accumulated in the transformants. Similarly, proteins related to oxidoreduction or lipid metabolism were down-accumulated. Other CAZy(s) acting on the fungal cell wall constituents (FCWE) were found among both down- and up-accumulated proteins. This remarkably shared profile between the transformants was strengthened by the distribution of the 149 differentially accumulated proteins that were common to NP/T1-4 (Figure 3A, black). Altogether these results suggest a common secretion defect in the 4 transformants.

To consolidate these data, the NP/T1-4 transformants were grown in CCPX liquid medium and the culture supernatants were collected to measure enzymatic activities (Figure 3B and Supplementary Table S6). On one hand, less than 10% of the parental activities were recorded for proteases, xylanases (hemicellulases), laccases, and lipases. On the other hand, cellulase and polygalacturonase activities were not significantly different when compared to the parental strain. These data are consistent with the proteomic results and indicate that the transformants are deficient in the secretion of several hydrolytic enzymes compared to the parental strain. In addition, the response of the transformants to oxidative stress, as well as their capacity to produce reactive oxygen species (ROS) were tested. When compared to the parental strain, no significant differences were observed in their responses to H_2_O_2_ or to menadione (data not shown). In contrast, oxidation of DAB was strongly affected in the four transformants (Figure 3C), indicating reduced H_2_O_2_ production. These results support the proteomic data showing down-accumulation of proteins related to oxidoreduction.

No GO enrichment analysis was performed for the 644 proteins likely secreted through non-conventional pathways (no SP and/or a TM domain) because no theoretical corresponding protein list can be extracted from the genome annotation data to run the enrichment calculation. *In silico* analysis supported the functional classification of 508 of these proteins (Supplementary Table S3) and highlighted 8 functional categories with up-accumulated proteins in all four transformants. Among the 81 proteins similarly accumulated in the four mutants, 64 were up-accumulated and 17 were down-accumulated. They were associated with metabolism, oxidoreduction, cytoskeleton, traffic, protein synthesis, protein homeostasis, signaling, and other CAZy (Supplementary Table S3).

### Transcriptomic Analysis of Transformants NP/T1-4 Under Plant-Mimicking Conditions

Understanding the virulence phenotype of transformants NP/T1-4 would have benefited from their investigation in contact with plants, but by being non-pathogenic, they could not produce the fungal material required for regular plant-fungus interaction studies. As a compromise, and based on data showing that plant-based culture media could trigger fungal behaviors that partly mimic those observed *in planta* (Billon-Grand et al., 2012), the transformants NP/T1-4 were grown in liquid medium containing cucumber leaves as unique nutritional source. The abundance of plant proteins in this medium excluded an analysis of the fungal exoproteome, but the mycelia of the transformants could be collected and used for RNAseq analysis.

**FIGURE 4:**
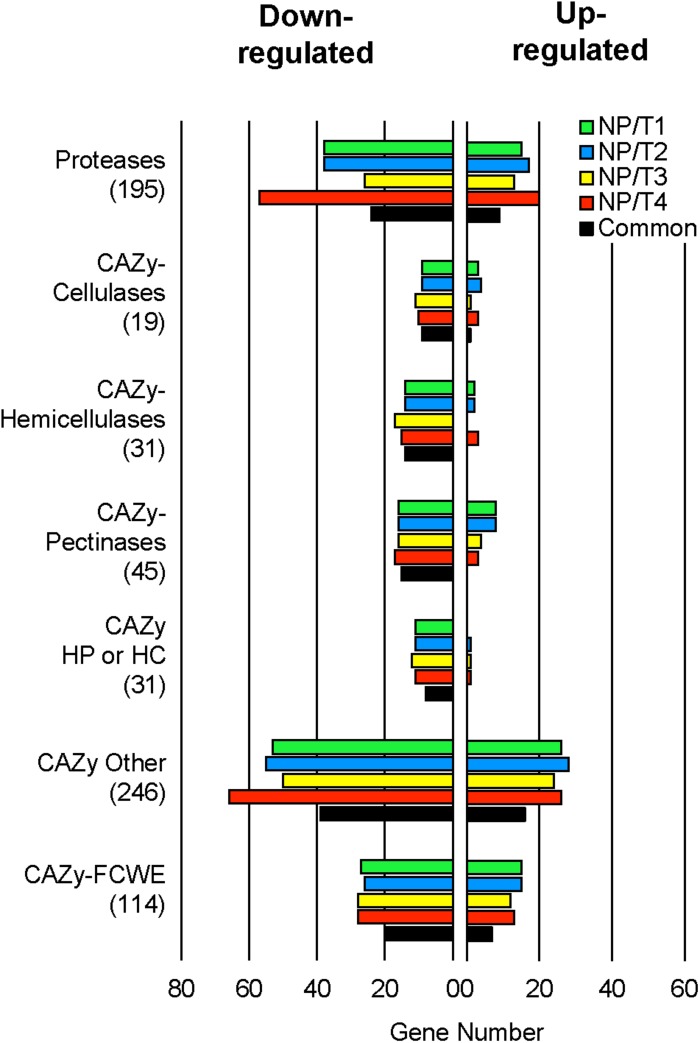
Transcriptomic analysis of transformants NP/T1-4 under plant-mimicking conditions. The transformants and the parental strain B05.10 were grown in liquid cucumber medium for 44 h and mycelia were collected for analyzing gene expression by RNAseq (Independent biological experiments *n* ≥ 3). Differentially Expressed (DE) genes (up- and down-) (see Supplementary Table S4), in comparison to the parental control (B05.10) for each transformant NP/T1-4, were classified in selected categories (Protease and CAZy) revealed by GO functional enrichment analysis. “Common” represents the DE genes present in all NP/T1-4. The total genes constitutive of each category are indicated in brackets. CAZy, carbohydrate active enzymes; HP, hemicellulose pectin; HC, hemicellulose cellulose; FCWE, fungal cell wall enzymes.

Among the 11,701 genes annotated in the B05.10 genome (van Kan et al., 2017), 1,911 genes (16%) were DE in at least one transformant when compared to the parental strain and, remarkably, 1,387 of these genes (72%) were DE in all 4 transformants (727 down-regulated and 660 up-regulated; Supplementary Table S4). GO functional enrichment analysis of the DE genes revealed no significant enrichment for the up-regulated genes whereas a significant enrichment in both carbohydrate metabolism and proteolysis were highlighted for the down-regulated genes as well as for the GO category « extracellular region » (data not shown). We observed that for each transformant 23–26% of the genes encoding proteins with a predicted SP and no TM domain were down-regulated and 11–13% up-regulated (Supplementary Table S4). We used the classification of CAZy to highlight the CAZy encoded by the DE genes. As shown in Figure 4, for each of the 4 transformants, a noticeable fraction (35–58%) of the genes coding cellulases, hemicellulases, pectinases and other CAZy cleaving hemicellulose and/or pectin and/or cellulose were down-regulated while a smaller fraction (0–21%) was up-regulated. Similarly, the fraction of down-regulated genes coding for other CAZy was larger (20–27%) than that of the up-regulated genes (10–11%). In parallel, the expression data revealed a similar profile for the protease- and the FCWE-encoding genes (Figure 4). When the common DE genes were analyzed, a similar general profile was obtained (Figure 4). Altogether these results indicate that there is a significant down-regulation of genes encoding secreted proteins in the 4 transformants, in particular of genes encoding plant cell wall-degrading enzymes (PCWDE).

### Comparison of the Proteomic and Transcriptomic Data

Results from the proteomic and transcriptomic approaches were compared to determine whether the DE genes in all four transformants corresponded to the differently accumulating proteins in the exoproteomes. This comparison showed that 63 (∼10%) of the 660 up-regulated genes and 160 (∼23%) of the 727 down-regulated genes, respectively encode proteins that are up- and down-accumulated in at least one transformant (Supplementary Table S5). These 160 proteins represent ∼52% of the down-accumulated proteins, 119 of them predicted with SP and 54 classified as CAZy. This result suggests that the down-accumulation of these enzymes in the NP/T1-4 exoproteome would be linked to reduced gene expression. Although the culture conditions were not identical (synthetic medium containing polymers and medium with gherkin cotyledons), the consistence between the proteomic and transcriptomic data obtained for the four mutants highlights a deficiency in secretion and more particularly in hydrolytic enzyme secretion.

### Impaired Growth on Specific Substrates in All 12 Non-pathogenic Transformants

**FIGURE 5:**
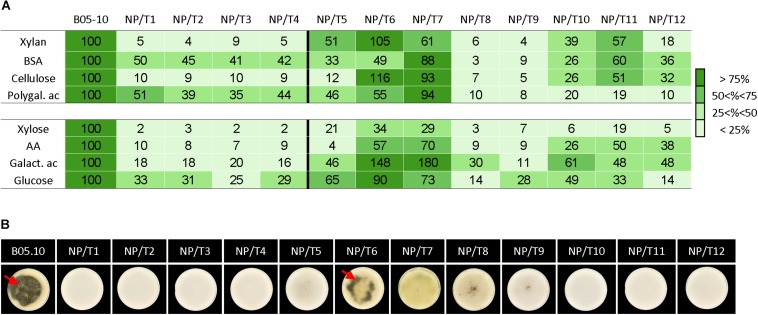
Growth on specific substrates and BSA degradation of transformants NT/T1-12. Parental strain B05.10 and transformants NP/T1-12 were grown on agarose 0.5% medium supplemented with various substrates (5 g/L) **(A)** and on solid BSA medium **(B)**. **(A)** The surface (mm^2^) covered by the mycelia was measured 4 day post-inoculation (dpi). Data are expressed as percentage of the parental control. The experiment was run in biological triplicates and the raw data are presented in Supplementary Table S7. BSA, Bovin Serum Albumin; Polygal. ac., Polygalacturonic acid; AA, Amino Acids; Galact. ac. Galacturonic acid. **(B)** BSA degradation by secreted proteases was revealed by translucent halos (red arrows) at 15 dpi. Pictures are representative of three independent biological experiments.

Although the NP/T1-4 strains grew similarly to the parental strain on complete medium, the data collected via the proteomic and transcriptomic approaches led us to hypothesize that these strains might grow slower than the parental strain on specific substrates. In consistence with the observed impaired xylanase and protease activities of the NP/T1-4 transformants, their radial growth rates on xylan and on bovine serum albumin (BSA) were reduced compared to the parental strain (>90% and 50–59%, respectively) (Figure 5A). In addition, no transparent halos were visible on solid BSA cultures of the transformants, indicating the absence of BSA degradation by secreted proteases (Figure 5B). On polygalacturonic acid or cellulose, the 4 transformants also grew slower than the parental strain (Figure 5 and Supplementary Table S7). This reduced growth was consistent with the transcriptomic data, but could not be predicted from the proteomic data (no significant differential accumulation between the transformants and the parental strain). Since an assimilation (transport and/or metabolism) default of the nutrients produced by these hydrolytic enzymes could explain the observed growth defect, the transformants were grown in presence of monosaccharides (xylose, galacturonic acid or glucose) or amino acids. As reported in Figure 5, the four transformants were impaired (70–80%) in their abilities to grow on these sugars or amino acids as substrates, indicating an impaired assimilation of these nutrients in all four transformants. Altogether these data demonstrate that multiple functions related to the degradation and/or nutritional assimilation of polymers are defective in the NP/T1-4 strains.

Lastly, the same specific substrates (polymers and corresponding monomers) were used to test the remaining 8 non-pathogenic transformants. The transformants NP/T8 and NP/T9 exhibited very affected growth profiles similar to those of the NP/T1-4 strains (all values below 40%). Four strains (NP/T5 and NP/10-12) exhibited moderately affected profiles and two strains (NP/T6-7) were rarely affected (Figure 5 and Supplementary Table S7).

### Identification of the Mutated Loci in the 12 Non-pathogenic Transformants

To identify the T-DNA integration events in the 12 non-pathogenic transformants, thermal asymmetrical interlaced (TAIL)-PCR was performed. The amplification of the T-DNA-flanking regions was unsuccessful in some cases, and therefore an alternative method was developed that rescued circularized T-DNA-containing genomic fragments to use them as PCR matrices. The latter technique allowed precise localization of the T-DNA in all transformants. According to that, the T-DNA is integrated in different loci, predominantly (in 10 transformants) in non-coding regions. For 5 transformants, the T-DNA insertion could be associated to a described gene (Supplementary Table S8). These genes encode ATPase subunit or proteins involved in signal transduction. Each T-DNA insertion was confirmed with locus-specific PCR primers. Furthermore, the parental loci were not amplified in 11 of the 12 transformants (data not shown), indicating that the transformants were homokaryotic.

## Discussion

Random mutagenesis based on ATMT has been extensively used in numerous fungi to identify novel genes involved in pathogenesis. The most extensive collection of random insertional mutants in a plant pathogenic fungus was generated by the *Magnaporthe oryzae* community and contains more than 70,000 transformants (Chen et al., 2011). In *B. cinerea* the same approach was developed and a first collection of 2,367 transformants was generated by P. Tudzynski’s Lab (Giesbert et al., 2012). The phenotyping and molecular characterization of these insertional mutants allowed the identification of several new virulence-related genes (Schumacher et al., 2019). Furthermore, two other collections of 800 and approximatively 5,000 ATMT-based mutants of *B. cinerea* have been generated more recently (Tayal et al., 2017; Liu et al., 2018). In all cases, the authors have used these libraries to identify proteins involved in virulence of the fungus. In this study, we present the generation of a new collection of 2,144 ATMT mutants of *B. cinerea*. The objective of this study was not only to extend the number of mutants altered in virulence and consequently the number of genes potentially involved in fungal virulence but also to precise if common signatures between non-pathogenic mutants could be observed.

The ATMT mutants generated in our library revealed several characteristics similar to those described in the two other collections of *B. cinerea*. Most of them (77% of the analyzed transformants) displayed a single and stable T-DNA integration as they were able to grow under selective pressure (hygromycin), even after several successive cultures without the selective agent. Moreover, the identification of the T-DNA integration sites using TAIL-PCR or plasmid rescue revealed that the insertions occurred in different genomic loci, preferentially in non-coding regions as previously reported in the other ATMT libraries (Choi et al., 2007; Bourras et al., 2012; Giesbert et al., 2012). As *B. cinerea* is a polyphageous pathogen, the transformants were tested on several host plants and 12 were non-pathogenic on cucumber cotyledons, primary bean leaves and apple fruits. Molecular characterization of these mutants showed that the T-DNA integrated in ORF only for two mutants. Among the different loci we identified, one of them (transformant NP/T2) was already studied and the deletion mutant was generated by Minz Dub et al. The characterization of this mutant revealed that the small GTPase BcRac was necessary for virulence (Minz Dub et al., 2013).

We considered exploiting these non-pathogenic strains to identify common traits linked with pathogenesis. *B. cinerea* is a typical necrotrophic fungus that secretes an arsenal of toxins including organic acids and plant cell wall-degrading enzymes to kill the host tissues and feed on the dead cells during colonization. To explore the different steps of the infection process from penetration to reproduction, we analyzed mycelial growth rates and the ability of the mutants to differentiate infection cushions (IC) and asexual reproduction structures (conidia and sclerotia). Their ability to acidify their environment and to secrete proteins, particularly enzymes involved in tissue maceration was analyzed as well.

The twelve non-pathogenic mutants shared common deficient traits such as significantly reduced conidiation and absence of sclerotial development. However, we cannot conclude that these traits are necessary for virulence as several mutants of the library do not produce conidia or sclerotia but are still fully virulent. The absence or alteration of IC formation seems to be a common trait that should be considered. Among the twelve mutants we analyzed, seven did not differentiate infection cushions and five exhibited an altered structure that did not further develop with time. This common trait we observed was also described in other mutants of *B. cinerea* with an altered virulence. For example, Hao et al. described two hypo-virulence-associated mycoviruses in *B. cinerea* whose interaction with the plant results in impaired IC formation (Hao et al., 2018), the deletion of *Bchbf1* (hyphal branching-related factor 1) results in smaller ICs compared to the wild-type strain (Liu et al., 2019) and the mutant of the aquaporin 8, considered as a critical component of *B. cinerea* virulence, does not differentiate IC(s) (An et al., 2016). In the necrotrophic fungus *Sclerotinia sclerotiorum*, the requirement of ICs for penetration and subsequent invasive growth is likewise important as shown for mutants deleted for the transcription factor SsNsd1 (Li et al., 2018) and the secretory Rhs repeat-containing protein SsRhs1 (Yu et al., 2017). All these results suggest the importance of this penetration structure for the virulence of these two necrotrophic pathogens.

Acidification of the ambient environment through the secretion of organic acids is an important aspect of the necrotrophic strategy of *B. cinerea* (Billon-Grand et al., 2012; Müller et al., 2018). To explore this point, the mutants were grown on a complete medium in presence of a pH indicator to reveal the medium acidification. In these experimental conditions, a defect in acidification was not observed for all the mutants, as only 5 of the 12 studied non-pathogenic mutants were deficient in acidifying their environment. However, we must consider these preliminary results with caution. Indeed, pH modulation was found to be tissue-dependent and results from the release of different organic acids including oxalic, citric, fumaric, succinic acids, and also ammonia (Billon-Grand et al., 2012; Müller et al., 2018). Quantification of these different metabolites should be precised in all the mutants as some of these metabolites seem to be particularly important for the virulence (Müller et al., 2018).

To investigate the molecular characterization of the mutants in depth, proteome and transcriptome analyses were performed on four mutants (NPT1/4) almost unaffected in hyphal growth but exhibiting defects in IC formation and medium acidification. The analysis of the exoproteome revealed a reduction of secreted proteins for all the mutants and a functional classification of these proteins disclosed common profiles between the four mutants. Interestingly, 115 common down-accumulated proteins were recorded in the four mutants and could be classified according to these common profiles. Significant variations focused on proteases and CAZy as more than 20 common proteases and 45 CAZy were down-accumulated in the mutants compared to the parental strain. These latter enzymes are known to play a major role in the degradation of the plant cell wall. Among these CAZy, hemicellulases were particularly affected as well as enzymes actives on other polysaccharides. The biochemical assays we performed validated these data as the mutants were severely affected in protease and xylanase activities. No obvious differences between the mutants and the parental strain were observed in the case of the pectinases and cellulases. Once again, the results of biochemical tests were in accordance with the proteomics data. Other enzymes participating to the plant tissue maceration such as cutinases or lipases (see Supplementary Table S3) were also found down-accumulated in the four transformants. Finally, we observed that more than 10 proteins related to oxidoreduction were down-accumulated in the four transformants. Among these proteins were recorded one tyrosinase, dioxygenases and the four laccases BcLCC2, BcLCC7, BcLCC9, and BcLCC10 (Supplementary Table S3). Biochemical tests revealing an alteration of the oxidation of DAB supported the down-accumulation of such proteins in the secretome of the mutants and quantification of the laccase activity confirmed the reduced activity of these enzymes.

The transcriptomics approach we performed on the four mutants partly confirmed the proteomic data and revealed a common molecular signature similar to those observed with the analysis of the exoproteome. In the plant-based culture media used in this study and designed to partly mimic the “*in planta*” conditions, 1,387 genes, corresponding to 11.8% of the genes of *B. cinerea* were deregulated in the four mutants we analyzed. As the mutants are non-pathogenic, it can be assumed that some of them, perhaps many, are involved in the virulence of the four mutants and potentially in the differentiation of the IC. GO functional enrichment analysis of the down-regulated genes revealed that genes involved in proteolysis and carbohydrate metabolism exhibited the most affected deregulation in the four mutants. We can suggest that they could contribute to elaborate a molecular hallmark of the non-pathogenic phenotype of *B. cinerea*. A focus on the expression of the genes encoding proteases and CAZy could be investigated in a first step as these genes appeared among the most deregulated in the mutants. Indeed, it has been reported that protease and xylanase-encoding genes were under-expressed in several other virulence-attenuated mutants of *B. cinerea* (Schulze Gronover et al., 2001, 2004; Schumacher et al., 2012, 2015; Müller et al., 2018). The molecular characterization of the other non-pathogenic mutants we isolated in the library should be performed in order to validate this hallmark. Finally, as we observed that genes encoding well-known virulence factors such as BcSp11 a cerato-platanin protein (Frias et al., 2011), aquaporin 8 (Aqp8) (An et al., 2016) or the xylanase11A (BcXyn11A) (Brito et al., 2006) were also under-expressed in the four mutants, these genes could also be considered in the establishment of the hallmark.

## Conclusion

In conclusion, we isolated several non-pathogenic mutants characterized by mutations in different loci of the genome and sharing common traits such as the absence or alteration of the IC, a reduced secretion of enzymes involved in the degradation of plant cell walls, e.g., hemicellulases and proteases and probably a defect in nutrient assimilation. These results also highlight the importance and the role of IC in the secretion of hydrolytic enzymes and beyond in the virulence of the pathogen. The investigation of the infection cushion should confirm that this structure is not only dedicated to penetration but also to the secretion of proteins required for virulence.

Finally, our study shows that random insertional mutagenesis is not only a strategy to identify new virulence factors but can also be used to precise developmental and molecular traits necessary for virulence, thus contributing to a better understanding of the infectious process.

## Data Availability Statement

The datasets generated for this study can be found in the Gene Expression Omnibus Database (GEO) GSE134112, PRIDE PXD013559.

## Author Contributions

GéM, MV, and NP designed the project. FV and M-PL contributed to the design and implementation of the project (generation of transformants and pathogenicity assays). LC, AV, PB, CD, SK, EL, CR, JS, and GuM conducted the experimental studies. CD and JD conducted the proteomic experiments. MC, CB, CD, ES, and NP analyzed the proteomic data. AS and MV analyzed the transcriptomic data. NP directed the project. AV supervised the students’ work. CB, AV, MV, JS, and NP wrote the manuscript.

## Conflict of Interest

LC, M-PL, and FV were employed by the company Bayer SAS. The remaining authors declare that the research was conducted in the absence of any commercial or financial relationships that could be construed as a potential conflict of interest.

## Footnotes

1 https://proteome.cgfb.u-bordeaux.fr/en

2 https://www.bioinformatics.babraham.ac.uk/projects/fastqc/

3 http://www.ncbi.nlm.nih.gov/geo/

4 http://www.cazy.org/

## Abbreviations

ATMT: agrobacterium tumefaciens mediated transformation
CAZy: carbohydrate active enzymes
DE: differentially expressed
FCWE: fungal cell wall enzymes
HC: hemicellulose cellulose
HP: hemicellulose pectin
SP: signal peptide
TAIL-PCR: thermal asymmetric interlaced polymerase chain reaction
TM: transmembrane domain
