# Supplementary material for: A Similar Secretome Disturbance as a Hallmark of Non-pathogenic Botrytis cinerea ATMT-Mutants?
Source: Front Microbiol. 2019 Dec 6;10:2829. doi: 10.3389/fmicb.2019.02829 (PMC6908482; doi:10.3389/fmicb.2019.02829)
Supplement: Supplementary file 1 [file Data_Sheet_1.PDF]

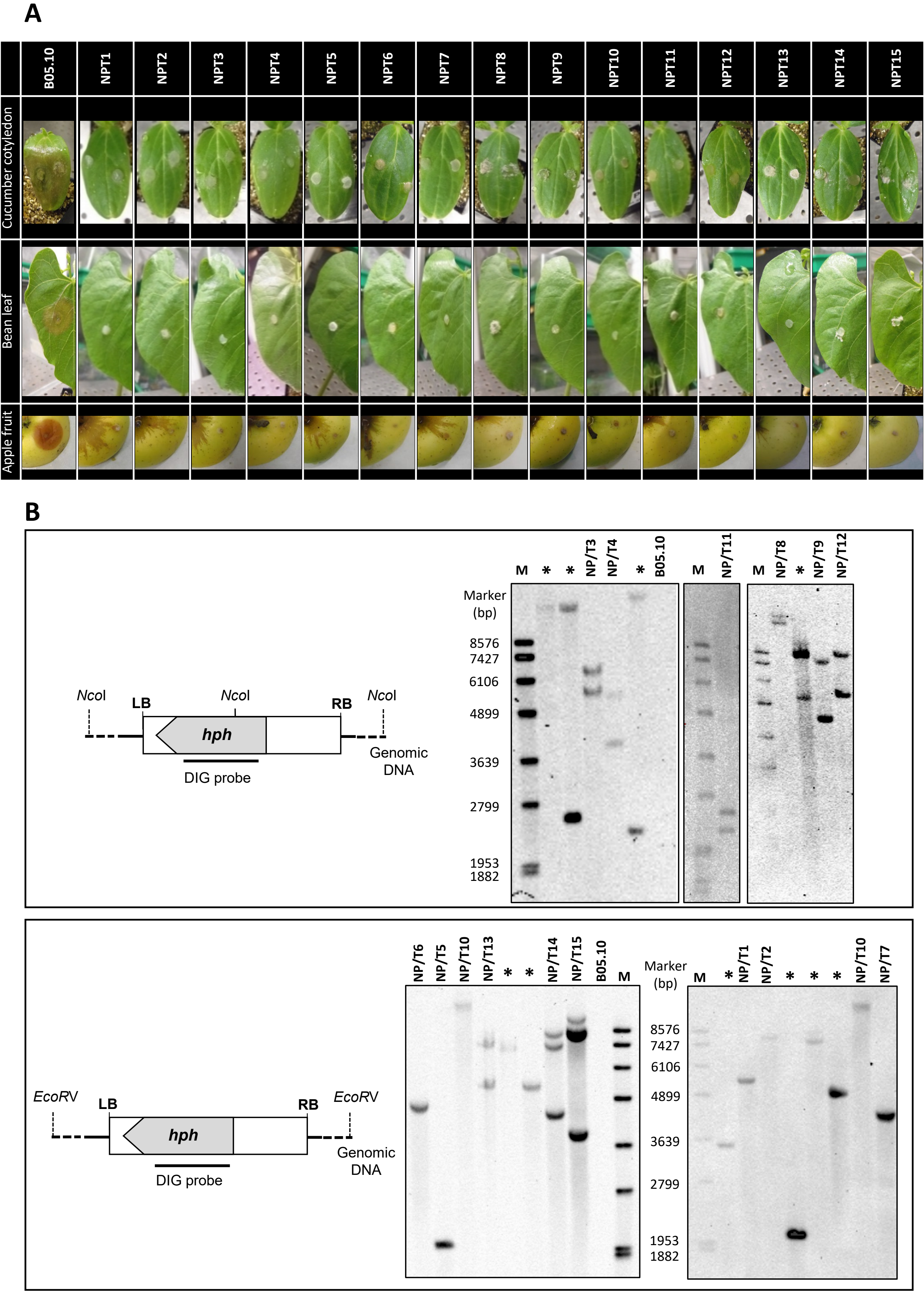

Figure S1: Virulence phenotype and Southern-blot analysis of 15 nonpathogenic transformants (NP/T1-15) of *B. cinerea*. A) The virulence of the *B. cinerea* ATMT transformants was compared to that of the parental strain B05.10 on cucumber cotyledons (*Cucumis sativus*, top), primary leaves of French bean (*Phaseolus vulgaris*, middle) and wounded apple fruits (var goldrush, bottom). Agar explants of non-conidiating mycelia were used as inoculum and pictures were taken 4 days post inoculation (dpi). Three independent experiments (≥48 inoculation points) were performed. **B)** Genomic DNA of the parental strain (B05.10) and transformants NP/T1-T15 digested with *Nco*I (top) or *EcoR*V (bottom) and probed with a digoxigenin (DIG)-labelled DNA fragment corresponding to the *hph* gene. NP/T1-12 show single T-DNA insertion and NP/T13-15 show multiple T-DNA insertions. (*) Mutant strains not retained in this study. LB, left border; RB, right border; M, DIG-labelled DNA marker.
